# Persistence and clearance rates of human papillomaviruses in a cohort of women treated or not treated for cervical dysplasia in northwest Ethiopia

**DOI:** 10.1038/s41598-025-87568-6

**Published:** 2025-03-10

**Authors:** Alemayehu Abate, Abaineh Munshea, Endalkachew Nibret, Dawit Hailu Alemayehu, Ashenafi Alemu, Alemseged Abdissa, Adane Mihret, Markos Abebe, Andargachew Mulu

**Affiliations:** 1 Health Biotechnology Division, Institute of Biotechnology, Bahir Dar University, P.O. Box 79, Bahir Dar, Ethiopia; 2 Medical Diagnostics Reference Laboratories Directorate, Amhara Public Health Institute, P.O. Box 477, Bahir Dar, Ethiopia; 3 Biology Department, College of Science, Bahir Dar University, P.O. Box 79, Bahir Dar, Ethiopia; 4 Armauer Hansen Research Institute, P.O. Box 1005, Addis Ababa, Ethiopia

**Keywords:** Biotechnology, Cancer, Microbiology

## Abstract

Persistence of high-risk Human papillomaviruses (HR-HPV) infection increases the risk of precancerous lesions development. The aim of this study was to assess the persistence and clearance rate of HPV infection. A prospective cohort study was conducted between January and December 2023 among patients attending gynecology unit of FHCSH in Bahir Dar, northwest Ethiopia. Out of 297 study participants, 95 women with HPV infected and cytological abnormalities were followed; of these 93.7% were HPV positive at the baseline study. Of which, 46.1% did not receive treatment, the rest 53.9% were treated. Among the women without treatment, HPV persistence and clearance rates were 65.9% and 34.1% respectively while persistence rate of 46.3% and clearance rate of 53.7% were observed in 12-month follow up period. Among women who received treatment, HPV persistence rate of 45.8% and clearance rate of 54.2% were recorded in six while persistence rate of 33.3% and clearance rate of 66.7% were observed in 12- month follow up period. The findings of our study indicated that the high persistence rate and low clearance rate of HPV infection. Detection of persistent HPV infection without treatment or after treatment should be considered as the main risk factor for the development or recurrence of cervical neoplasia.

## Introduction

Infections of Human papillomaviruses (HPVs) frequently spread through sexual contact. These viruses have the ability to infect squamous epithelial cells found in the skin and mucosa^1,2^. These viruses, including 40 human infecting different varieties, can lead to cervical cancer by infecting the mucous membranes lining the uterus and transforming neighboring cells into malignant ones^3^. Although most cervical HPV infections are transient, some people experience persistent HPV infections. In middle-aged women, persistent high risk (HR) HPV, infection is strongly linked to both precancerous lesions and cervical cancer. Persistent HPV infections are those that result in positive findings from two consecutive HPV tests that are spaced at least six months apart^4^.

Cervical intraepithelial neoplasia (CIN) is a protracted phase of cytological alterations that typically precedes cervical cancer and takes 15 to 20 years before the invasive carcinoma manifests. Therefore, if cellular alterations are identified and treated at an early stage, cervical cancer can be prevented^5^. With appropriate follow-up and treatment, premalignant lesions can be treated to prevent them from developing into in-situ or invasive cancer^6^.

The recommended therapy for high-grade cervical intraepithelial neoplasia (CIN) based on histological diagnosis is ablative or excisional treatment to stop the disease’s development to cervical cancer. But even after excisional surgeries, 10–53% of women may still have the disease^7^.

In most industrialized nations where timely treatment, high-quality screening, and follow-up care services are consistently provided, cervical cancer rates have decreased^8^. The bulk of cases and fatalities occur in low- and middle-income countries (LMICs), where progress in reducing incidence and mortality has been poor. Several countries have reported rises in incidence or mortality rates in the last ten years^9,10^.

Precancerous lesions and cervical cancer are mostly caused by persistent infections with specific types of the high-risk human papillomavirus^11,12^. Most HPV-infected women typically clear the virus within 6–12 months, while the likelihood and time-to-viral clearance may vary depending on factors such as the women’s age, HPV type, sexual behavior, and treatment status at baseline^13^.

About 70% of HPV infections resolve spontaneously within one year, and 90% in two years; the virus still exists in the remaining cases. An efficient cell-mediated immune response is necessary for HPV elimination. As a result, HIV-positive people who contract HPV are more likely to develop benign warts and malignant tumors because they are less likely to clear the infections between one and two years^14^.

Currently, our understanding of the natural history, carcinogenic characteristics, screening, and preventative strategies of HPV infection is comparatively clear. Nonetheless, HPV infection rates are high, particularly in underdeveloped nations where the incidence and prevalence of cervical cancer are still high. Low socioeconomic standing, a lack of population knowledge, and poorly executed screening and immunization programs are some of the causes^15^.

Prior research has demonstrated that regional and national differences in HPV incidence, persistence, and clearance^16^. The aim of this study was to assess the persistence and clearance rate of HPV infection among women who received treatment and not treated for cervical dysplasia.

## Results

In this study, a total of 324 study participants were enrolled in the study but 27 of them had insufficient PAP smears and they were excluded from the study, the rest 297 were included, HPV DNA or VIA or PAP positive or CIN II^+^women (113/297) from the baseline assessment were included in the follow up study, of which 18/113 women (15.93%) were lost during follow up. The major reason to follow up was change of contact details which made the patients to be unreachable, referred to an oncology center for advanced treatment and death. A total of 95 women were followed, 89/95 was HPV positive in the baseline study. Of which 41/89 were not treated due to only HPV positive, LSIL for PAP and VIA positive but not eligible for treatment according to the WHO classification. However, the rest 48/89 women were treated. Majority of study participants (75.3%; 67/89) were age between 30 and 50 years old. The details of socio-demographic, sexual and reproductive characteristics are presented in Table 1.

**Table 1:** Socio-demographic, sexual and reproductive characteristics of study participants included in the follow up study.

Variables	Categories	Follow up (*n* = 89)	Percentage
Age (year)	< 30	10	11.2%
	30–50	67	75.3%
	> 50	12	13.5%
Educational status	Illiterate	76	85.4%
	Read and write	2	2.2%
	Elementary	4	4.5%
	High school and above	7	7.9%
Monthly income	< 2000 birr	23	25.8%
	2001–5000 birr	33	37.1%
	> 5000 birr	33	37.1%
Marital status	Single	15	16.9%
	Married	49	55.1%
	Divorced	9	10.1%
	Widowed	16	18%
Parity	< 3	19	21.3%
	4–6	50	56.2%
	> 6	20	22.5%
Occupation	Employed	31	34.8%
	Others	58	65.2%
Residence	Urban	36	40.4%
	Rural	53	59.6%
Age at first marriage	< 18	38	42.7%
	18–20	27	30.3%
	> 20	24	27%
Age at first sexual debut	< 18	41	46.1%
	18–20	32	36%
	> 20	16	18%
Number of lifetime sexual partners	≥ 2	68	76.4%
	1	21	23.6%
Number of current sexual partners	≥ 2	74	83.1%
	1	15	16.9%
Condom during sexual intercourse.	No	51	57.3%
	Yes	38	42.7%
Hormonal contraceptive use > 5 years	No	59	66.3%
	Yes	30	33.7%
Personal hygiene	No	65	73%
	Yes	24	27%
Have you heard about cervical cancer	No	48	53.9%
	Yes	41	46.1%
Have you been screened before	No	62	69.7%
	Yes	27	30.3%
Co-existing medical condition	No	66	74.2%
	Yes	23	25.8%
Family history of cervical cancer	No	80	89.9%
	Yes	9	10.1%
History of STI infection	No	35	39.3%
	Yes	54	60.7%
HIV status	Positive	17	19.1%
	Negative	72	80.9%
CD4 count	< 500 cells/mm3	13	14.6%
	501–999 cells/mm3	62	69.7%
	≥ 1000 cells/mm3	14	15.7%
HPV Infection	Sin gle	58	65.17%
	Multiple	31	34.8%

### HPV persistent rate and clearance rate among non-treatment group

Among the non-treatment group, HPV persistence rate of 65.9% (27/41) and clearance rate of 34.1% (14/41) were recorded in 6 month follow up time at (95% CI 0.190–0.493, *P* < 0.001) while persistence rate of 46.3% (19/41) and clearance rate of 53.7% (22/41) were observed in 12 month follow up period at (95% CI: 0.377–0.696, *P* < 0.001).

HPV-16 had high persistent rate 92.31%(12/13) among women who were not treated in 6 month follow up period, and its clearance rate was 7.69%(1/13), and at 12 month follow up period, the persistent rate of 76.92%(10/13) and clearance rate of 23.08%(3/13) were recorded at (95% CI:0.034-0.496, *P* = 0.082). The persistent rate of multiple infection was 84.6%(11/13) at 6 month follow up, were higher than the persistent rate of single infection (57.1%(16/28). The persistent rate of multiple infection at 12 month was 76.9%(10/13) which was also higher than the persistent rate of single infection(32.1%(9/28)). The details are presented in Tables 2 and 3.

**Table 2:** HPV persistence and clearance rate at six-month follow up without treatment stratified by HPV type.

HPV genotypes	Frequency	Baseline	6 months Persistent and clearance rate without treatment			
			Persistent rate	Clearance rate	95% CI	*P*-value
High and probable high risk	89	41	65.9%(27/41)	34.1%(14/41)	0.190–0.493	< 0.001
Type-16	40	13	92.31%(12/13)	7.69%(1/13)	-0.09-0.25	0.337
Type 16 alone	24	8	87.5%(7/8)	12.5%(1/8)	-0.17-0.42	0.351
Type-18	16	7	71.43%(5/7)	28.57%(2/7)	-0.17-0.74	0.172
Type-35	12	6	50%(3/6)	50%(3/6)	-0.08-1.08	0.076
Type-52	12	4	75%(3/4)	25%(1/4)	-0.55-1.05	0.391
Type-58	18	8	75%(6/8)	25%(2/8)	-0.14-0.64	0.170
Other high risks (HPV-31/33/39/45/51/56/59/66/68	24	15	60%(9/15)	40%(6/15)	0.12–0.68	< 0.001
Possible and low risk	31	21	71.4%(15/21)	28.6%(6/21)	0.08–0.49	0.01
Type-53	16	9	66.67%(6/9)	33.33%(3/9)	-0.05-0.72	0.081
Type-70	8	6	66.67%(4/6)	33.33%(2/6)	-0.02-0.88	0.175
Other possible and low risks (HPV-6/11/26/40/42/44/54/61/73/82)	16	13	69.23%(9/13)	30.77%(4/13)	0.02–0.59	0.04
Single	58	28	57.1%(16/28)	42.9%(12/18)	0.23–0.62	< 0.001
Multiple	31	13	84.6%(11/13)	15.4%(2/13)	-0.07-0.38	0.165

**Table 3:** HPV persistence and clearance rate at 12-month follow up without treatment stratified by HPV type.

HPV genotypes	Frequency	Baseline	12 months Persistent and clearance rate without treatment			
			Persistent rate	Clearance rate	95% CI	*P*-value
High and probable high risk	89	41	46.34%(19/41)	53.7%(22/41)	0.377–0.696	< 0.001
Type-16	40	13	76.92%(10/13)	23.08%(3/13)	-0.03-0.49	0.082
Type 16 alone	24	8	62.5%(5/8)	37.5%(3/8)	-0.06-0.81	0.08
Type-18	16	7	57.14%(4/7)	42.9%(3/7)	-0.07-0.92	0.078
Type-35	12	6	33.33%(2/6)	66.67%(4/6)	0.13–1.21	0.025
Type-52	12	4	75%(3/4)	25%(1/4)	-0.55-1.05	0.391
Type-58	18	8	50%(4/8)	50%(4/8)	0.05–0.95	0.033
Other high risks (HPV-31/33/39/45/51/56/59/66/68	24	15	40%(6/15)	60%(9/15)	0.32–0.88	< 0.001
Possible and low risk	31	21	47.62%(10/21)	52.4%(11/21)	0.29–0.76	< 0.001
Type-53	16	9	44.44%(4/9)	55.6%(5/9)	0.15–0.96	0.013
Type-70	8	6	50%(3/6)	50%(3/6)	-0.08-1.08	0.076
Other possible and low risks (HPV-6/11/26/40/42/44/54/61/73/82)	16	13	53.85%(7/13)	46.15%(6/13)	0.15–0.78	< 0.001
Single	58	28	32.1%(9/28)	67.9%(19/28)	0.49–0.86	< 0.01
Multiple	31	13	76.9%(10/13)	23.1%(3/13)	-0.03-0.49	0.082

### HPV persistence rate and clearance rate among treatment group

Among women who received treatment, persistence rate of 45.8% (22/48) and clearance rate of 54.2% (26/48) were observed in 6 month follow up period at (95% CI: 0.395–0.688), *P* < 0.001), persistence rate of 33.3% (16/48) and clearance rate of 66.7% (32/48) were observed in 12 month follow up period at (95% CI: 0.1528–0.805), *P* < 0.001).

At 6 month follow up period after treatment, other high risk HPVs (HPV-31/33/39/45/51/56/59/66/68) had the highest persistence rate of 66.67%(6/9) and low clearance rate of 33.33%(3/9) with (95%CI: -0.51-0.718, *P* = 0.081), followed by HPV-52, HPV-18 and HPV-16 with persistent rate of 62.5%(5/8), 55.59%(5/9), 51.85%(14/27) and clearance rate of 37.5%(3/8) with (95%CI:0.058–0.808, *P* = 0.081), 44.44%(4/6) with (95%CI:0.039–0.850, *P* = 0.035) and 48.15%(13/27) at (95% CI: 0.280–0.683, *P* < 0.001).

At 12 month follow up period after treatment, HPV-18 had the highest persistent rate of 44.44%(4/9) and clearance rate of 55.56%(5/9) with (95%CI: 0.150–0.961, *P* = 0.013), followed by other high risk HPVs, HPV-16 and HPV58 with persistence rate of 44.44%(4/9), 40.74%(11/27), 30%(3/10) and clearance rate of 55.56%(5/9) with (95%CI: 0.150–0.961, *P* = 0.013), 59.26%(16/27) with (95%CI:0.359–0.791, *P* < 0.001), and 70%(7/10) with (95% CI:0.354–1.046, *P* < 0.001).

The persistent rate of multiple infection was 61.1%(11/18)at 6 month follow up, were higher than the persistent rate of single infection 36.7%(11/30). The persistent rate of multiple infection at 12 month was 38.8%(7/18) which was also higher than the persistent rate of single infection(30%(9/30)). The details are presented in Tables 4 and 5.

**Table 4:** HPV persistence and clearance rate at six-month follow up after-treatment stratified by HPV type.

HPV genotypes	Frequency	6- month persistent and clearance rate after treatment				
		Baseline	Persistent	Clearance	95% CI	*P*-value
High and probable high risk	89	48	45.83%(22/48)	54.17%(26/48)	0.39–0.69	< 0.001
Type-16	40	27	51.85%(14/27)	48.15%(13/27)	0.28–0.68	< 0.001
Type 16 alone	24	16	31.25%(5/16)	68.75%(11/16)	0.43–0.94	< 0.001
Type-18	16	9	55.56%(5/9)	44.44%(4/9)	0.04–0.85	0.035
Type-35	12	6	33.33%(2/6)	66.67%(4/6)	0.13–1.21	0.025
Type-52	12	8	62.5%(5/8)	37.5%(3/8)	-0.06-0.81	0.08
Type-58	18	10	40%(4/10)	60%(6/10)	0.23–0.97	< 0.001
Other high risks (HPV-31/33/39/45/51/56/59/66/68	24	9	66.67%(6/9)	33.33%(3/9)	-0.51-0.72	0.081
Possible and low risk	31	10	50%(5/10)	50%(5/10)	0.12–0.88	0.015
Type-53	16	7	42.86%(3/7)	57.14%(4/7)	0.08–1.07	0.03
Other possible and low risks (HPV-6/11/26/40/42/44/54/61/70/73/82)	24	4	25%(1/4)	75%(3/4)	-0.05-1.55	0.058
Single	58	30	36.7%(11/30)	63.3%(19/30)	0.45–0.82	< 0.001
Multiple	31	18	61.1%(11/18)	38.9%(7/18)	0.12–0.46	0.01

**Table 5:** HPV persistence and clearance rate at 12- month follow up after-treatment stratified by HPV type.

HPV genotypes	Frequency	Persistent and clearance rate after treatment at 12-month follow up				
		Baseline	Persistent	Clearance	95% CI	*P*-value
High and probable high risk	89	48	33.33%(16/48)	66.7%(32/48)	0.53–0.81	< 0.001
Type-16	40	27	40.74%(11/27)	59.26%(16/27)	0.39–0.79	< 0.001
Type 16 alone	24	16	31.25%(5/16)	68.75%(11/16)	0.43–0.94	< 0.001
Type-18	16	9	44.44%(4/9)	55.56%(5/9)	0.15–0.96	0.013
Type-35	12	6	16.67%(1/6)	83.33%(5/6)	0.41–1.26	< 0.001
Type-52	12	8	25%(2/8)	75%(6/8)	0.36–1.14	< 0.001
Type-58	18	10	30%(3/10)	70%(7/10)	0.35–1.05	< 0.001
Other high risks (HPV-31/33/39/45/51/56/59/66/68	24	9	44.44%(4/9)	55.56%(5/9)	0.15–0.96	0.013
Possible and low risk	31	10	30%(3/10)	70%(7/10)	0.35–1.05	< 0.001
Type-53	16	7	14.29%(1/7)	85.71%(6/7)	0.14–0.51	< 0.001
Other possible and low risks (HPV-6/11/26/40/42/44/54/61/70/73/82)	24	4	25%(1/4)	75%(3/4)	-0.05-1.55	0.058
Single	58	30	30%(9/30)	70%(21/30)	0.53–0.87	< 0.001
Multiple	31	18	38.8%(7/18)	61.1%(11/18)	0.36–0.86	< 0.001

### Comparison of HPV persistent infection rate between treatment and non-treatment group

**Fig. 1:**
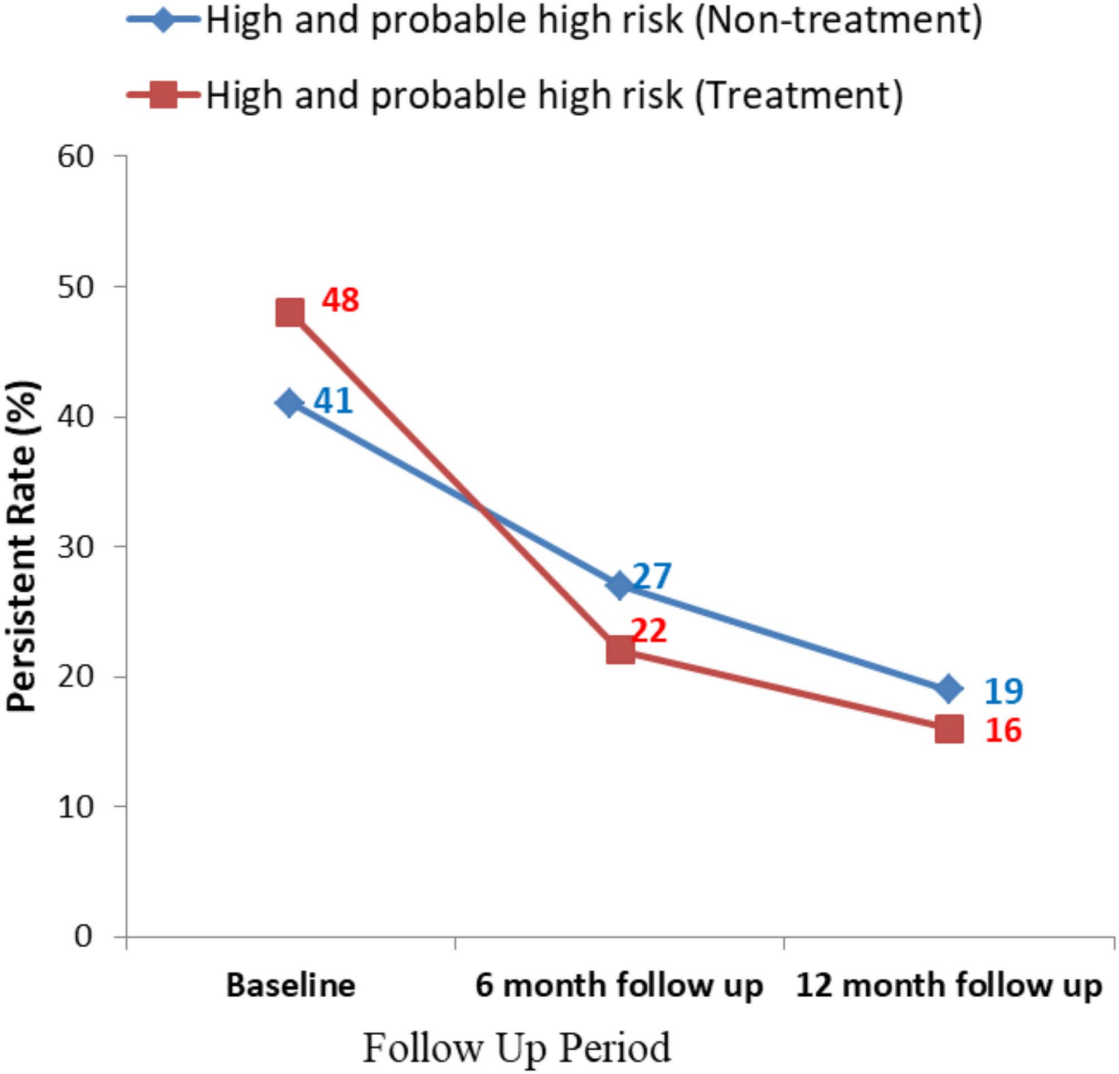
Comparison of high risk and probable high risk HPV persistent infection rate between treatment and non-treatment group.

**Fig. 2:**
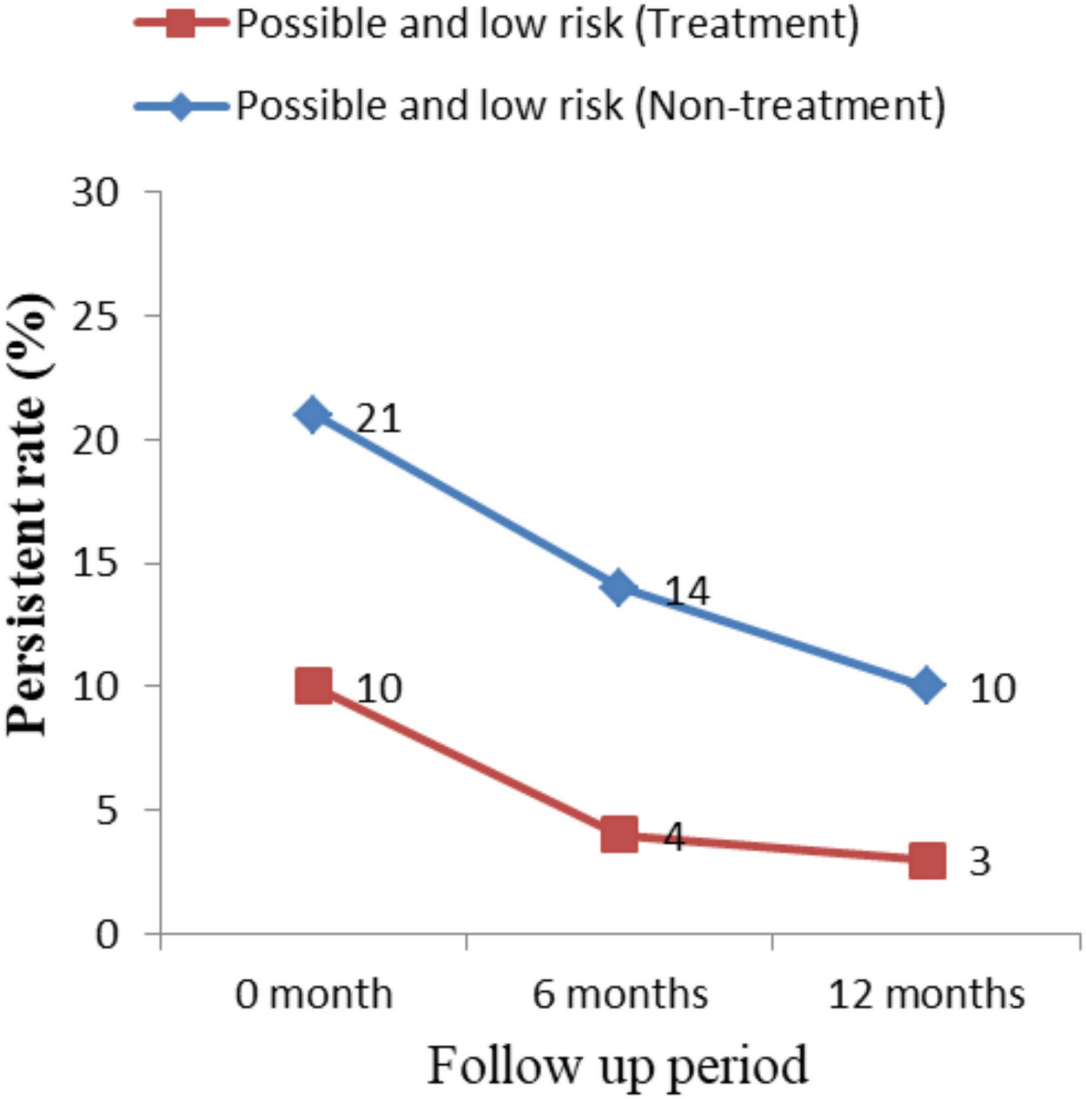
Comparison of persistent infection rate of possible high risk and low risk HPV between treatment and non-treatment group.

The rate of persistence shows some differences between treatment group and non-treatment group. At 12 month, the persistent rate of high and probable high risk genotypes in treatment group was 33.33%, whereas the persistent rate of non-treatment group was 46.34% (Fig. 1). At 12 month, the persistent rate of possible and low risk genotypes in treatment group was 30%, whereas the persistent rate of non-treatment group was 47.62% (Fig. 2).

## Discussion

To our knowledge, the present study is the first to examine the persistence and clearance rate of HPV infection in women who received treatment and untreated for cervical dysplasia. HPV positive women in the baseline study were followed for one year with a six month interval. Persistent infection of HPV will lead to development of precancerous lesions and cervical cancer. Since women treated for cervical cancer and precancerous lesions are at risk of disease recurrence, monitoring of HPV infection at follow-up is crucial. The objective of the current prospective study was to determine the persistence rate and clearance rate of HPV infection at six and twelve months among women without treatment and women who received treatment.

In the present study, among women without treatment, persistence rate of 65.9% and clearance rate of 34.1% were recorded in 6 month follow up period, persistence rate of 46.3% (19/41) and clearance rate of 53.7% (22/41) were observed in 12 month follow up period. This finding is consistent with a study conducted in Zimbabwe, which found that the rates of persistence and clearance were 34.6% and 65.4%, respectively^17^, . Our results also almost similar to a Ugandan study that found 31.2% of women completely clearing their infections^18^.

However our finding is low compared to a Brazilian study that found 40.4% and 59.6% of women, respectively, experienced viral infection clearance or persistence^19^. A Chinese study reported that, of 298 HPV-positive patients with CIN1 or normal cervical histology at baseline, 120 (40.26%) had persistent infection whereas 178 (59.74%) cleared their infection after a year^20^. The observed difference might be attributed to variations in the studies’ follow-up periods, sample sizes, and recruitment of women with normal and cervical squamous intraepithelial lesions (SILs).

In the current study, HPV-16 had high persistent rate of (92.31%; 12/13) among women without treatment in six- month follow up whereas its clearance rate was low (7.69%; 1/13). However at 12 month follow up, the persistence rate was 76.92%(10/13) while clearance rate was 23.08%(3/13). HPV-52 had by persistent rate of 75%(3/4) and clearance rate of 25%(1/4). HPV 18 had persistent rate of 57.14%(4/7) and clearance rate of 42.9%(3/7) at 12 month follow up period comparable with a Zimbabwean study which indicated HPV-16 was the most persistent type (53.8%), HPV-52 was the second most persistent type^17^.

In this study, among women who were treated, persistence rate of 45.8% (22/48) and clearance rate of 54.2% (26/48) were recorded in 6 month follow up period., persistence rate of 33.3% (16/48) and clearance rate of 66.7% (32/48) were observed in 12 months, highly consistent with a Korean study indicated that following cold knife conization or loop electrosurgical excision treatment, fifty-eight patients (33.7%) had HPV infection^17^. This result is also consistent with findings from other Korean studies that indicate from 398 patients, 28 (30.2%) had persistence after conization or Loop electrosurgical excision procedure (LEEP)^21^, and 48 (30%) had persistent HR HPV infections and 112 (70.7%) had HPV cleared following therapy^22^. However, this result is greater than that of an Italian study that found 96 individuals (23.5%) continued to have at least one genotype following therapy^23^.

In the present study, HPV-18 had the highest persistent rate 44.44%(4/9) among treated women in 12 month follow up and its clearance rate was 55.56%(5/9), followed by other high risk HPVs, HPV-16 and HPV58 with persistent rate of 44.44%(4/9), 40.74%(11/27), 30%(3/10) and clearance rate of 55.56%(5/9), 59.26%(16/27), and 70%(7/10), similar with a Turkish study which indicated HPV-18 was cleared in almost all (95.8%) cases, followed by HPV 16 (69.9%) and other HR HPV types (65.6%)^7^. However other studies found that the persistent infection rate for HPV-16 was higher than that of other HPV strains^17,24,25^.

In our study, the persistent rate had decreased from six month to twelve month in both women who were not treated (from 65.9 to 46.34%) and women who were treated (from 45.83 to 33.33%). This result is supported by a systematic review by Hoffman et al.^26^ which reported that median HPV persistence tended to decline over time: 27% at three-month, 21% at six-month and 10% at 24 -month after treatment. Additionally, they stated that a variety of factors, including the patient’s age, the type of HPV, the method of detection, the course of treatment, and the minimum interval between HPV post-treatment tests, affected the persistence of post-treatment HPV. The result of the current study indicted there is a need to follow HPV positive women for the persistent infection and development of cervical abnormalities or recurrence of cervical neoplasia, which is supported by a study conducted in the United States that found women with persistent HPV infection had a higher risk of developing abnormalities in their epithelial cells^27^.

## Conclusion

Our study contributes to the knowledge of persistence rate and clearance rate of HPV infection among women who received treatment and untreated for cervical dysplasia. There were high persistence rate and low clearance rate of HPV infection among women who received treatment and untreated for cervical dysplasia in Amhara regional state. Detection of persistent HPV infection among women who received treatment and untreated for cervical dysplasia should be considered as the main risk factor for the development or recurrence of cervical neoplasia. Our results confirmed the clinical impact of HPV genotyping for more frequent follow up and on management and post-treatment surveillance of cervical neoplasia. Further larger-scale with longer period studies with minimum interval between HPV tests are necessary for the better understanding of the persistence rate and clearance rate of HPV infections and development or recurrence of precancerous lesions and cervical cancer.

## Methods

### Study setting and population

A prospective cohort study was conducted among patients attending gynecology unit of FHCSH in Bahir Dar, northwest Ethiopia, between January and December 2023. A total of 297 women were enrolled in the study. All study participants were screened with Visual Acetic Acid (VIA) test, genotyping of the HPV DNA and cervical cytology examination with PAP test. Study participants with positive results from the screening tests were examined with colposcopy and biopsy was taken for histopathology. Two follow-up visits were scheduled at 6 and 12 months after the baseline visit. After the result of baseline diagnosis, a total of 95 of 297women were followed, 89/95 were HPV positive in the baseline study, were followed for persistence and clearance of HPV infection at 6 and 12 month period.

### Study population

The study population was all women of age above 30 and HIV positive women with all age range who visited FHCSH Gynecological unit and suspected for cervical cancer.

### Sample size determination

Study one: A single population proportion sample size determination formula (study population size less than 10,000 ^32^) was used with the assumption of 19% proportion of, 5% margin of error, and 95% desired level of confidence interval and considering a 10% non- response rate, the sample size was 281.

Study two: Sample size was determined from formulation of sensitivity and specificity test using Power Analysis and Sample Size (PASS) software based on desired type I error, power and effect size ^33^. The minimum sample size was determined by taking the prevalence of a disease 19%, by assuming sensitivity of the kit is comparable with the gold standard, and specificity of the kit is greater than 70%, the power is set to be at least 80% and the p-value, is set to be less than 0.05. The sample size for sensitivity and specificity of Onco E6 performance study was 49 positive for histopathology, 196 negative samples with the gold standard histopathology examination and 49 negative controls. By taking 10% contingency, the sample size was 324. The final sample size was the highest sample size of the upper studies, 324 sample size was determined for this study.

### Sampling technique and procedure

The sampling technique was systematic random sampling. Every third study participants was selected after a random starting study participant who was selected by lottery method.

### Follow up visits

All study participants were screened with Visual acetic acid test (VIA), genotyping of the HPV DNA test and cervical cytology examination with PAP test. Study participants with positive results from screening tests were examined with colposcopy and biopsy was taken for histopathology. Participants with positive results of the screening or diagnostic tests were followed. Two follow-up visits were scheduled 6 and 12 months after the baseline visit. Women with positive results for VIA, PAP smear and histopathology were treated with thermocoagulation, Loop Electrosurgical Excision Procedure (LEEP) and cryotherapy.

### Specimen collection

Data were collected at three time points during the study: baseline, 6-month follow-up and 12-month follow-up. At the baseline visit, upon arrival at the cervical cancer unit, trained gynecologists currently working on cervical cancer unit conducted physical and gynecological examination, screen with VIA and collect swab for HPV DNA test, smear for cytologic specimens. Tissue biopsy was collected for all positive study participants from the screening tests when there were positive results of colposcopic impression. Swab specimens were collected using PreservCyt solution (Halogic.Inc., Marlborough MA, USA) following the manufacturer’s instructions for collecting and handling cervical specimens in preservCyt solution. The study procedures were explained to each study participants and written informed consent was obtained from those who agreed to participate. Treatment was given for women with positive for VIA or HSIL^+^ for PAP or CIN II^+^ for histopathology.

### HPV genotyping and histopathology examinations

HPV genotyping was performed to detect high-risk HPV DNA in cervical swabs. DNA extraction, amplification and HR-HPV partial genotyping was performed using the Abbott Real Time HR-HPV assay (Abbott Molecular, Des Plaines, IL, USA). This is an automated process that uses micro beads technology for DNA extraction. The assay is a qualitative in-vitro test that amplifies and detects HR-HPV DNA in cervical cells. Detection of all 14 h-HPV genotypes (HPV 16, 18, 31, 33, 35, 39, 45, 51, 52, 56, 58, 59, 66, and 68) was achieved through a primer mix targeting the conserved L1 region of HR-HPV genomes and single stranded DNA probes^28^.Then extended genotyping was performed by Anyplex™ II PCR System to detect human papillomavirus DNA in cervical swabs, based on a real-time multiplex PCR assay that allows simultaneous amplification, detection, and differentiation of target nucleic acids of 19 high-risk HPV types (-16, -18, -26, -31, -33, -35, -39, -45, -51, -52, -53, -56, -58, -59, -66, -68, -69, -73, -82) and 9 low-risk HPV types (-6, -11, -40, -42, -43, -44, -54, -61, -70)^29^.

Biopsies were independently examined by two experienced pathologists. When the diagnosis differed between the two pathologists, the sample was reviewed by a third pathologist and consensus obtained. Histo-pathological diagnosis had confirmed test results as Negative for Dysplasia/malignancy, CIN I, CIN II, CIN III, and cervical cancer cases.

### Statistical analysis

Statistical analyses were performed using SPSS version 26.0. Descriptive statistics such as frequency and cross tabulation were performed to summarize the data. Proportion difference at six and twelve months from baseline proportion was analyzed for persistent and clearance of infection. Persistent infection was defined as positive test results for specific HPV genotype detected at 6 months and 12 months tests. Clearance of HPV infection was defined as the first negative test result at any follow-up after a baseline positive test result for the specific genotype. HPV persistence and clearance rates were calculated for individual HPV types. Proportion was calculated using Z-test. Crude Odds Ratio (COR) and Adjusted Odds Ratio (AOR) at 95% confidence interval were calculated to assess the degree of association between the odds of persistence and clearance rate of specific HPV type among women who received treatment and the odds of persistence and clearance rate of specific HPV type among women who did not receive treatment (Considered as reference category in logistic regression). Finally, variables with a p-value of less than 0.05 were considered statistically significant.

## Data Availability

All the generated data in this article are included in the manuscript. Theoriginal data can be obtained from the principal investigator upon request. Alemayehu Abate alexu2love@gmail.com.

## Abbreviations

DNA: Deoxyribonucleic acid
HPV: Human Papilloma virus
HR-HPV: High-risk human Papilloma virus
CI: Confidence interval
STIs: Sexually transmitted Infections
CIN: Cervical intraepithelial neoplasia
ICC: Invasive cervical cancer
PCR: Polymerase chain reaction
